# Superior sagittal sinus thrombosis presenting as a continuous headache: a case report and review of the literature

**DOI:** 10.1186/1757-1626-2-9361

**Published:** 2009-12-21

**Authors:** Rishi K Gupta, Aimun AB Jamjoom, Upendra P Devkota

**Affiliations:** 1 Nottingham University Medical School, Queens Medical Centre, Nottingham, UK; 2 Department of Neurosurgery, National Institute of Neurological and Allied Sciences, Kathmandu, Nepal

## Abstract

Cerebral venous sinus thrombosis is a rare but dangerous condition, occurring with an incidence of 3-4 cases/million/year. Cerebral venous sinus thrombosis presents a diagnostic challenge due to its varied presentation patterns. We report a case of a 42 year old Nepali man diagnosed with cerebral venous sinus thrombosis after presenting with a week long history of continuous headache. He improved rapidly following prompt anticoagulation. Despite thorough investigation no discernable underlying cause could be found. Our report highlights the value of prompt diagnosis of cerebral venous sinus thrombosis through neuroimaging and the importance of immediate anticoagulation as part of patient management.

## Introduction

Cerebral venous sinus thrombosis (CVST) is an uncommon condition affecting 3-4 cases/million/year with a mean age of 37 to 38, though any age may be affected [1,2]. Women tend to be at an increased risk particularly between the ages of 20 - 35, mainly due to the use of the oral contraceptive pill and the post partum state [1,3]. Predisposing risk factors can be identified in up to 80% of patients [4]. The clinical presentation of CVST is varied and can include headache, vomiting and seizures. This variation creates a diagnostic challenge for clinicians.

In this article we report a patient who developed a superior sagittal sinus thrombosis with no discernable underlying cause. It is hoped that the presentation of this case will draw attention to this rare and potentially devastating disease; helping increase clinicians' awareness of the clinical characteristics, methods of diagnosis and management of CVST.

## Case presentation

**Figure 1:**
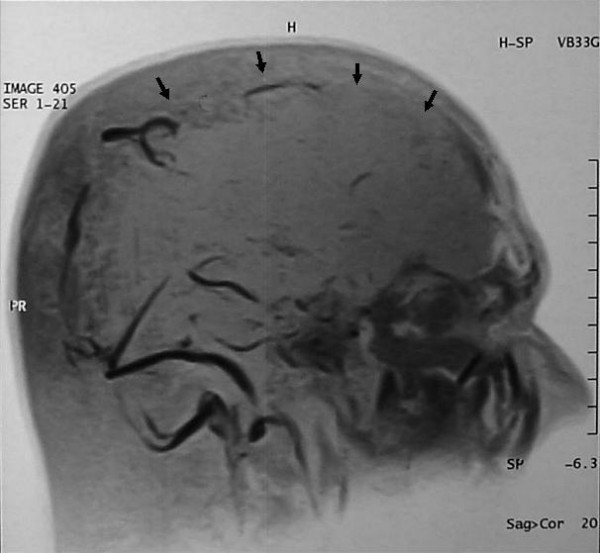
**MRV 2 days post admission demonstrating thrombosis of the superior saggital sinus (arrows)**.

**Figure 2:**
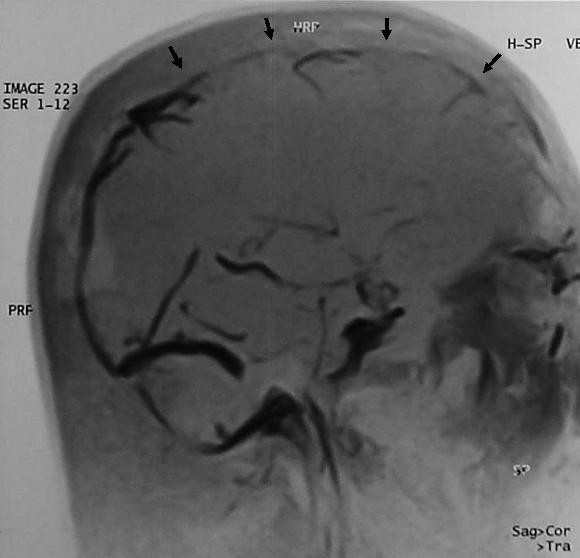
**MRV 8 days post diagnosis showing improved perfusion of the superior sagittal sinus following anticoagulation (arrows)**.

A 42-year old Nepali male was admitted to the National Institute of Neurological and Allied Sciences, Kathmandu complaining of a week-long history of a continuous headache. The headache was global in nature, with no associated vomiting. The evening preceding admission, the patient had also noted photophobia, speech disturbance and weakness of his right upper limb. There was no history of trauma, no relevant past medical or drug history and no family history of note. The patient did however mention partaking in unaccustomed strenuous activity earlier in the week. On examination, the patient's observations were stable with a GCS of 15/15. His pupils, fundi and speech appeared normal and there was no evidence of any focal neurological deficit or meningism. A computerized tomography (CT) scan appeared normal and lumbar puncture showed an opening pressure of 30 cm H_2_O with normal constituents. D-dimer and fibrinogen degradation product (FDP) were marginally raised; all other parameters were within normal ranges. Magnetic resonance venography (MRV) confirmed the presence of a superior sagittal sinus thrombosis with a small venous infarct (Figure 1) and the patient was subsequently anticoagulated with enoxaparin and warfarin. Further investigations including echocardiography, carotid artery Doppler, coagulation studies and antiphospholipid antibody titres were all normal. A follow-up MRV was performed 8 days after diagnosis which showed improved perfusion throughout the superior sagittal sinus (Figure 2). The patient made a good recovery and was subsequently discharged home 10 days post admission.

## Discussion

Venous drainage of the brain involves blood flow through cerebral veins into the dural sinuses that then drain into the internal jugular vein. As in our reported case, the superior sagittal sinus is the main cerebral venous sinus affected by thrombus and is involved in 62% of cases [5]. The underlying pathogenesis of CVST involves two mechanisms. The first includes localized oedema and venous infarction due to cerebral vein occlusion. The second mechanism constitutes the development of raised intracranial pressure due to occlusion of one of the cerebral venous sinuses, since CSF absorbed by the arachnoid villi drains into the superior sagittal sinus [5]. Our patient demonstrated signs of both these mechanisms. However, it has been shown that, in a fifth of cases, only raised intracranial pressure occurs with no signs of cortical vein thrombosis [6].

Predisposing risk factors are found in 80% [4]. This may include prothrombotic conditions [4], head injury [7], inflammatory disease [8], dehydration [9] and malignancy [6,9]. After extensive investigation, our patient demonstrated no underlying risk factors associated with CVST. Given that the patient lives in Kathmandu which lies 1324 m above sea level we can tentatively speculate that this high altitude may have had a hand in the formation of the CVST as this has been demonstrated in a number of previous cases [10,11]. It should be mentioned however, that patient suffering from thrombophilic states have been shown to be at particular risk of developing CVST at high altitude [12,13]. Institutional experience suggests that CVST can represent an aspect of high altitude illness and should be included as a differential for patients presenting with high altitude cerebral oedema [unpublished]. Further to this, the patient describes unaccustomed strenuous exercise prior to development of his headache which may indicate that dehydration may have also played a part in the development of his CVST.

The variation in which CVST presents makes it a diagnostic challenge. Our reported patient presented with a primary complaint of continuous headache which occurs in over 90% of cases [5]. Symptoms tend to develop insidiously over days to weeks [2]. However, sudden onset with a thunderclap headache has been reported [14]. Rarely, a unilateral focal neurological lesion such as hemiparesis may develop however this typically becomes bilateral in the following few days. Seizures occur in 40% of patients and act as a helpful differentiator between venous and arterial thromboses [5]. Raised intracranial pressure can lead to visual disturbances and papilloedema on fundoscopy [15].

Investigations are used in this condition in confirming the diagnosis, and establishing any predisposing causes. Initially, conventional CT scanning is usually performed to exclude other possible diagnoses such as subarachnoid haemorrhage. In 35% of published cases, CT scanning with contrast enhancement may demonstrate an 'empty delta sign' which is a useful radiological sign for the diagnosis of superior sagittal sinus thrombosis [16]. Furthermore, the use of transcranial color-coded duplex sonography (TCCS) is becoming increasingly common as a complement to other imaging techniques in the diagnosis of CVST [17]. Other initial investigations may include lumbar puncture, which has been found to be abnormal in 84% of cases of CVST, and may also be helpful in ruling out other diagnoses. The CSF abnormalities noted in our patient (raised opening pressure) are well-recognized in CVST, along with increased protein content and pleocytosis [18]. The preferred and most sensitive diagnostic investigation is magnetic resonance venography (MRV) [19] which allows the venous occlusion to be identified along with any consequences such as cerebral oedema and areas of venous infarction, as was present in our patient's case. It has been suggested that CT venography detects CVST with equal accuracy and may be advantageous in allowing a prompt diagnosis immediately after the initial CT scan [20]. If doubt remains following MRV, invasive cerebral angiography may be performed.

Initial management of patients with confirmed CVST should include stabilization and the prevention of cerebral herniation. This may involve the use of Mannitol or surgery [5]. Despite the risk of haemorrhage into venous infarcts, anticoagulation forms the mainstay of treatment, even in the presence of an existing haemorrhagic venous infarct [21], thus heparin is usually given after confirmation of the diagnosis. A prospective study found that 79% of patients recovered with this treatment [6]. Our patient showed marked clinical and radiological improvement following administration. Warfarin is usually continued for 6 months, or longer in the presence of predisposing conditions, with a target INR above 2.5 in order to reduce the risk of recurrent cerebral and extracranial thromboses. In cases where prognosis appears poor, endovascular thrombolysis, with agents such as urokinase and tissue plasminogen activator, may be used [5]. Other treatment options include the use of therapeutic lumbar punctures, oral acetazolamide and surgery in treating intracranial hypertension.

The prognosis of CVST is usually favourable but is worse in extremes of age, those with underlying conditions such as malignancy and sepsis, and in the presence of coma or deep cortical venous thrombosis [4]. More than 80% of patients, as in our case, have a good neurological outcome [5].

## Conclusion

CVST is a challenging condition due to its wide range of clinical presentations. Clinicians should have a high index of suspicion, even in the absence of predisposing conditions, in order to facilitate a prompt diagnosis through neuroimaging. Expedited standard management for the CVST should be employed to help ensure the best possible outcome for patients.

## Abbreviations

CSF: cerebrospinal fluid; CT: computed tomography; CVST: cerebral venous sinus thrombosis; FDP: fibrinogen degradation product; GCS: Glasgow Coma Scale; MRV: magnetic resonance venography; TCCS: transcranial color-coded duplex sonography.

## Competing interests

The authors declare that they have no competing interests.

## Authors' contributions

RKG helped gather information regarding the patient and complete the manuscript. AABJ helped gather information regarding the patient and complete the manuscript. UPD supervised the case report and reviewed the final manuscript. All authors read and approved the final manuscript.

## Consent

Written informed consent was obtained from the patient for publication of this case report and accompanying images. A copy of the written consent is available for review by the journal's Editor-in-Chief.
